# Can Students’ Computer Programming Learning Motivation and Effectiveness Be Enhanced by Learning Python Language? A Multi-Group Analysis

**DOI:** 10.3389/fpsyg.2020.600814

**Published:** 2021-01-21

**Authors:** Hsiao-Chi Ling, Kuo-Lun Hsiao, Wen-Chiao Hsu

**Affiliations:** ^1^Department of Marketing, Kainan University, Taoyuan City, Taiwan; ^2^Department of Information Management, National Taichung University of Science and Technology, Taichung, Taiwan

**Keywords:** Python, learning motivation, computer programming self-efficacy, maladaptive cognition, learning performance

## Abstract

Python language has become the most popular computer language. Python is widely adopted in computer courses. However, Python language’s effects on the college and university students’ learning performance, motivations, computer programming self-efficacy, and maladaptive cognition have still not been widely examined. The main objective of this study is to explore the effects of learning Python on students’ programming learning. The junior students of two classes in a college are the research participants. One class was taught Java language and the other class was taught Python language. The learning performance, motivations, and maladaptive cognition in the two classes were compared to evaluate the differences. The results showed that the motivations, computer programming self-efficacy, and maladaptive cognition on the learning performance were significant in the Python class. The results and findings of this study can be used in Python course arrangement and development.

## Introduction

With the rapid development of artificial intelligence (AI)-related technology, the demand for relevant talents is also increasing (Wu et al., 2020). This has caused a rise in global programming education. More than 25 countries have formulated relevant education policies to cultivate people’s programming skills at a young age. Those policies’ purpose is to cultivate talents in related industries and improve a country’s overall innovation and competitiveness by training people to have better logical and computational thinking. Consequently, programming-related courses have inevitably become essential (Kong et al., 2020).

Although programming is a necessary critical skill for students majoring in information technology-related disciplines, their learning performance of programming might be affected due to the possible maladaptive cognition in the process of their learning programming (Piwek and Savage, 2020). There are many kinds of programming languages; if students cannot master fundamental programming skills, they would have worse learning performance when learning other programming languages in the future.

The debate on determining which programming language a novice chooses has been ongoing (Pears et al., 2007). If a beginner chooses a complex language when he first enters the field, it is easy to lose interest in the programming field. According to reports from the IEEE (Institute of Electrical and Electronics Engineers), both Java and Python have been in the top-ten-list of programming languages in recent years. For example, the top programming language in 2015 was Java (IEEE Spectrum, 2015), and in the 2018 survey, Python became the top one (IEEE Spectrum, 2018). Khoirom et al. (2020) compared various characteristics and features of Java and Python. However, they did not discuss the differences in learning motivation and learning effects of beginners in learning two languages.

Python is undoubtedly the most popular among the AI-related programming languages because its programming syntax is relatively simple, it has a gentle learning curve, and its applications are diversified and broad (Gorelick and Ozsvald, 2020). Using Python can usually quickly develop simple and practical applications without spending much time, which can improve learners’ sense of accomplishment and is suitable for beginners. Nevertheless, few studies explore whether learning Python can better improve learners’ learning performance compared with other programming languages. Therefore, this study aims to explore whether, compared with learning Java, students who learn Python are more likely to improve their learning motivation and self-efficacy, reduce their maladaptive cognition, and improve their learning achievement of programming.

Python is one of the most popular languages in teaching introductory programming courses. However, the impact of learning Python on students’ learning performance has not been widely studied. In this study, students’ learning motivation and self-efficacy are regarded as influencing factors. The purpose is to explore whether the influencing factors would affect their learning achievement and whether maladaptive cognition might occur in the process of their programming learning courses. The research questions are as the following: (1) Will students’ learning motivation be higher while learning the Python programming language? (2) Will students’ learning performance and self-efficacy be better while learning the Python programming language? (3) Will students’ maladaptive cognition be lower while learning the Python programming language?

The main contributions of this paper are listed below:

(1) This study verified that after students learn Python language, their learning motivation and computer program self-efficacy were significantly improved.
(2) This study verified that their maladaptive cognition significantly decreased after learning Python.
(3) The students who learned Python and those who learned Java were compared in the experiments. The results showed that the former group had better learning performance.

It is expected that the results of this study will provide teachers with a reference for teaching programming courses. To cultivate students’ programming skills, in addition to strengthening students’ logical thinking, different programming languages might be used to enhance students’ learning motivation and self-efficacy and to reduce their maladaptive cognition in the process of their programming learning.

## Literature Review and Hypotheses

### Differences Between Python and Java

A survey report in The State of Developer Ecosystem 2020 pointed out that Java is the most popular primary programming language, although Python has overtaken Java (Jet Brains, 2020). More and more respondents are choosing to use Python to develop programs. The PYPL (PopularitY of Programming Language Index), which is created by analyzing how often language tutorials are searched on Google, found that Python grew the most in the last five years (19.1%) and surpassed Java in 2018. Due to the above facts, the literature was stimulated to explore the differences between Python and Java (Carbonnelle, 2020).

Miller (1956) believes that one of the most significant differences between Python and Java is how variables are handled. Java uses static typing, which forces programmers to define the variable’s type when they declare it for the first time. In contrast, Python uses dynamic typing, which allows programmers to change the type of a variable. For novice programmers, dynamic typing is more comfortable to master. However, many developers believe that static typing can reduce the risk of undetected errors that can cause issues for the program (Scanlan, 1989; Prechelt and Unger, 1999).

Ogbuokiri et al. (2016) compared Python and Java to find out which is the most suitable for teaching beginners in computer programming. The research experiment compared the differences between the two languages in terms of runtime or execution time, memory consumption, code size/program length, correctness/robustness, amount of comments, and reliability. Experimental results showed that Python consumes less memory and code size than Java. Moreover, Python executes faster and is more robust than Java. Based on the above results, although the study recommended that Python be used in the first course of computer programming courses for novices, there was no evidence that learning python is better than learning Java in terms of motivation and effectiveness.

Khoirom et al. (2020) analyzed and compared the features, advantages, and disadvantages of Java and Python. It was also mentioned that Python and Java are easy to learn, and there is significant work opportunity for developers in both fields. The study’s conclusions include that Java is more complicated in structure than Python, but it is easier for developers to understand memory management. Because Python is written in simple English, the syntax is short and easy to use. It is easier for novices to understand the program. The study focuses on comparing the syntax of the two languages. It is not about the psychological aspects of learners in learning two languages.

### Learning Motivation

People’s actions, desires, and needs are all derived from motivation to drive people’s behaviors or arouse someone’s desire to do something (Elliot and Covington, 2001; Vansteenkiste et al., 2020). Learning motivation is the degree of students’ willingness to continue to learn hard (Wang et al., 2020). Motivation is one of the primary factors that drive students to learn. Past studies showed that students’ learning motivation impacts teaching results (Law et al., 2019; Sanaie et al., 2019). El-Adl and Alkharusi (2020) also believed that learning motivation is the motivation of achievement for learners to maintain their learning activities in their learning processes. It is one of the critical factors affecting learning performance (Rocha et al., 2019; Gan, 2020).

The ARCS model of motivation proposed by Keller and Reigeluth (1983) is based on the systematic design model of stimulating students’ students’ motivation to integrate the motivation model derived from motivation theory and the related theories. Proposing the ARCS motivational design model in 1984, Keller believed that four key factors affect learning motivation, namely, attention, relevance, confidence, and satisfaction. The purpose is to help curriculum design or improve teaching, and it was emphasized that these four factors should be utilized to stimulate learners’ learners’ learning. Keller also believed that the ARCS motivational design model is suitable for learners of all ages.

Python programming language offers simple-to-learn syntax and a large standard library. The advantages can attract beginners to learn and enhance their confidence and satisfaction. Therefore, the following hypothesis is proposed.

H1: Students’ learning motivation can be significantly enhanced by learning the Python language.

### Maladaptive Cognition

Learning strategy is also known as a cognitive strategy used to explain an individual’s control of his/her learning, memory, and thinking behavior (Biwer et al., 2020). In past studies, the adaptive cognitive learning strategy was categorized as deep information processing strategy (organization and elaboration strategies) and self-adjustment strategy (observation, judgment, and response).

Scholars such as Pintrich and DeGroot also categorize cognitive strategy as rehearsal strategy, elaboration strategy, and organization strategy. Rehearsal strategy was considered as a surface cognitive strategy (Anderman and Young, 1994; Bandura, 1997), through which learning content can stay in the short-term memory for a short time. Surface cognitive strategy, also known as surface strategy, is regarded as a passive cognitive strategy (Entwistle and Tait, 1990).

Surface strategy is a strategy in which learners use repetitive rehearsal to keep learning content in their short-term memory and avoid it being forgotten quickly. If learners frequently use this way to learn, they tend to lack in-depth thinking in their learning process, resulting in their inability to organize, integrate, absorb, and internalize the learning content. Consequently, in this study, maladaptive cognition is defined as the degree of using surface strategy to learn.

The syntax of Python language is ease-of-learning, so the learners can keep the syntax in their long-term memory. They also can concentrate their attention on the coding logic, not on the syntax. Therefore, the following hypothesis is proposed.

H2: Students’ maladaptive cognition will be lower while learning the Python language.

### Self-Efficacy and Learning Achievement of Programming

Self-efficacy refers to one’s degree of belief in whether one can use his/her skills to complete a particular behavior (Bandura, 1986). Korkmaz and Altun (2014) used to apply the “Computer Programming Self-Efficacy Scale” to 378 engineering students to test students’ self-efficacy levels for learning C++ programming language. The study results showed that the scale has trustworthiness, reliability, and validity, which can be used to quantify the self-efficacy perception of engineering students. It was also found that computer engineering students’ self-efficacy is higher than that of electronic engineering students. Many studies in the past have also shown that students’ self-efficacy would significantly affect their learning achievement.

Learning achievement is defined hereby as learners’ achievement during their programming learning course, and is used to evaluate whether learners’ programming skill is improved upon completing their course. In this study, students’ final examination results are used as the evaluation index of their programming learning achievement. Learning achievement is a critical index to evaluate students’ learning outcomes. Tanah (2009) believed that learning achievement is the degree of learners’ mastery of teaching materials, while the factors that affect learning achievement were also explored in past studies.

Python language has user-friendly data structures that can reduce the length of code. Learners can finish their computer programming tasks more quickly and easily. They also can complete their programming tasks through open source code in the Python online communities. Their computer programming self-efficacy and achievement will be enhanced by the advantages of Python language. Hence, the following hypotheses are proposed.

H3: Students’ computer programming self-efficacy can be enhanced significantly while learning the Python language.
H4: Students’ learning achievement can be enhanced significantly while learning the Python language.

## Research Method

The junior students of two classes in a college in central Taiwan are the research participants. The major of the students is information management. In this study, the questionnaire survey method is used. In the first semester, the students of class A were taught with Java. The pre-learning questionnaires for the students were collected at the beginning of the semester, and the post-learning questionnaires for the same students were again collected at the end of the semester after they learned Java. Pearson correlation analysis was further performed against sample data of students’ post-learning questionnaires and their final examination results to understand their learning performance in Java. In the second semester, the students of class B were taught with Python. The pre-learning questionnaires for the students were collected at the beginning of the semester, and the post-learning questionnaires for the same students were again collected at the end of the semester after they learned Python. Sample data of students’ pre- and post-learning questionnaires and the two classes’ final examination results were analyzed to understand the difference in students’ learning performance in Python programming language between the two classes. In addition to the programming language, we try to control the other conditions, such as the same class settings, instructor, and the same test. Before this study, a programming test was used to evaluate students’ programming abilities. The test results also showed that there were no significant ability differences in these two classes.

### Questionnaire Development

A five-point Likert scale was used for all items, ranging from “strongly disagree” (1) to “strongly agree” (5). The items for learning motivation were adapted from Keller (2009). Items for measuring computer programming self-efficacy were adapted from Ramalingam and Wiedenbeck (1998). Maladaptive cognition was assessed by the items developed based on the operational definition. A pretest was performed with the help of six students with computer programming experience and three experts.

### Validity and Reliability

In this study, SmartPLS 2.0, AMOS 22, and SPSS 24.0 were used to analyze the survey data. In the measurement model, confirmatory factor analysis (CFA) and exploratory factor analysis (EFA) were used to examine the reliability and validity of the model. All of the factor loadings in the factor analysis are greater than 0.7. The model fit index of the 3-factor CFA model are above acceptable values (GFI = 0.93, CFI = 0.99, AGFI = 0.85, TLI = 0.98, RMSEA = 0.066). With regard to the reliability, composite reliability (CR) and Cronbach’s alpha (CA) are the common criteria. The values of CR and CA ranged from 0.755 to 0.960, which exceed the 0.6 threshold for acceptable reliability (Esbensen, 2009). Analysis of the measurement model indicates the following: all items’ indicator factor loadings exceeded the accepted reliability threshold of 0.5, average variance extracted (AVE) values were within the range of acceptability (0.662–0.889), and all values for CR exceeded the accepted threshold. All the figures in the measurement model meet the conditions for convergent validity (Fornell and Larcker, 1981). With regard to discriminant validity, it commonly measures the statistical difference between two factors by comparing each construct’s square root of AVE with that construct’s correlation coefficients with the remaining constructs. The analysis results show that the correlation coefficients are less than the square root of the AVE. Hence, the results of the discriminant validity are acceptable.

## Results

In order to evaluate the learning performance in the two classes, paired sample *t*-tests were conducted to compare the students’ perceptions of the pretest with the perceptions of the post-test. In class B (Table 1), the perceptions of learning motivation and computer programming self-efficacy were enhanced significantly after learning Python (*p* < 0.05). The students’ maladaptive cognition was lower and improved significantly (*p* < 0.01). In class A (Table 2), students’ perceptions of learning motivation were not enhanced after learning the Java language. Surprisingly, their computer programming self-efficacy and maladaptive cognition were not significantly improved either (*p* > 0.05).

**TABLE 1:** Paired *t*-test in Python language course (*N* = 35).

Construct		Mean	SD	*t*	*df*	*p*
LM	Pretest	3.50	0.79	–1.795	34	**0.082**
	Post-test	3.70	0.74			
MC	Pretest	2.71	0.99	3.024	34	**0.005**
	Post-test	2.29	1.04			
CPSE	Pretest	3.20	0.92	–2.517	34	**0.017**
	Post-test	3.55	0.78			

*LM, learning motivation; MC, maladaptive cognition; CPSE, computer programming self-efficacy. Bold values indicate the probabilities of observing the test results under the null hypotheses.*

**TABLE 2:** Paired *t*-test in Java language course (*N* = 34).

Construct		Mean	SD	*t*	*df*	*p*
LM	Pretest	3.28	0.80	2.910	33	0.006
	Post-test	2.91	0.81			
MC	Pretest	3.13	0.84	0.671	33	0.507
	Post-test	3.05	0.85			
CPSE	Pretest	3.09	0.75	–1.339	33	0.190
	Post-test	3.25	0.91			

*LM, learning motivation; MC, maladaptive cognition; CPSE, computer programming self-efficacy.*

In order to compare the differences in the learning performance in the two classes, a two-sample *t*-test is used to test the differences. The students’ learning performance was measured by a final programming exam, which contains six coding tests. According to the results in Table 3, there were no significant differences in learning motivation, computer programming self-efficacy, and maladaptive cognition between the two classes before this experiment. As expected, after this experiment, the students’ learning performances in the Python class is significantly better than the performances in theJava class. Therefore, H1-H4 were supported.

**TABLE 3:** Independent *t*-test of the two courses.

	Mean (SD)		*t*-value	*p*-value	Hypothesis testing
	Python	Java			
	(*N* = 35)	(*N* = 34)			
LM	3.50 (0.79)	3.28 (0.80)	1.149	0.255	
MC	2.71 (0.99)	3.12 (0.84)	–1.861	0.067	
CPSE	3.20 (0.92)	3.09 (0.75)	0.550	0.584	
LM (post)	3.81 (0.89)	2.91 (0.81)	4.379	**0.000**	Supported
MC (post)	2.31 (0.93)	3.05 (0.85)	–3.412	**0.001**	Supported
CPSE (post)	3.71 (0.90)	3.25 (0.91)	2.111	**0.038**	Supported
LP (post)	91.43 (14.38)	73.53 (25.57)	3.570	**0.001**	Supported

*LM, learning motivation; MC, maladaptive cognition; CPSE, computer programming self-efficacy; LP, learning performance. Bold values indicate the probabilities of observing the test results under the null hypotheses.*

The results in the correlation test show that students’ scores in the Python class were positively related to the other three factors (Table 4). However, students’ scores in the Java class had no significant relationship with the three factors. The possible reasons may be the scores in Java were relatively low, and Java language is relatively hard to learn; students need more time to develop better learning performance.

**TABLE 4:** Correlation analysis.

Python	1	2	3
1. LM	–	–	–
2. MC	−0.605**	–	–
3. CPSE	0.680**	−0.687**	–
4. LP	0.501**	−0.818**	0.541**

**Java**	**1**	**2**	**3**

1. LM	–	–	–
2. MC	−0.333*	–	–
3. CPSE	0.748**	−0.374*	–
4. LP	0.287	0.029	0.121

*p < 0.05, **p < 0.01.

*LM, learning motivation; MC, maladaptive cognition; CPSE, computer programming self-efficacy; LP, learning performance.*

## Discussion

According to the data analysis results of this study, whether students learn Java or Python, there is a significant positive correlation between students’ learning motivation and self-efficacy of programming, which is also justified by a past study that stated that learning motivation would affect self-efficacy (Wang et al., 2008). In addition, learning motivation and maladaptive cognitions are shown to be significantly negatively correlated for students’ learning of Java and Python, which indicates that, with better learning motivation, students would obtain a sense of achievement from learning the programming language, and are willing to continue to participate in their learning; they would also have better self-efficacy perception of programming, increase their own level of confidence, lower the degree of their maladaptive cognition, and become more willing to learn the programming language through understanding rather than memorizing by rote.

The data analysis results show a significant positive correlation between students’ maladaptive cognition and self-efficacy of programming, whether students learn Java or Python. It indicates that having completed homework or tests by rote memorization, students’ confidence in coding a complete program to achieve a particular goal would become lower, and their self-efficacy worse.

In terms of different programming language courses, this study’s research results show that students would reduce their maladaptive cognition by learning Python. In other words, students in the Python class would improve and complete the course objectives not by rote memorization and instead would gradually learn and complete the course objectives with the intent of mastering the course in a comprehensive manner. However, after that, students in the Java class seem to not have a significant improvement in their maladaptive cognition. In other words, compared with the Java course, students tend to reduce their maladaptive cognition more by learning Python.

Past studies used to point out that there is a correlation between learners’ self-efficacy perception and their learning achievements. A good learning experience can improve one’s own self-efficacy and bring better learning achievement. According to the data analysis results of this study, there is a significant positive correlation between self-efficacy of programming and learning achievement of programming in the process of learning Python, although the foregoing significant correlation is not seen in the process of learning Java. It can be concluded that the higher the student’s self-efficacy perception of programming during students’ Python learning, the higher their confidence in their skill to code is, and thereby the better their learning achievements would be.

In terms of different programming language courses, the research results of this study show that students learning Python would increase their confidence in coding a program to achieve their task goals when learning Python, but students learning Java would not significantly improve their self-efficacy when learning Java, which indicates that for students, learning Python would improve their self-efficacy more against that achieved through learning Java.

According to the analysis results of the Pearson correlation test, students’ learning motivation, maladaptive cognitions, self-efficacy of programming, and learning achievement of programming all show a significant correlation to one another for the students leaning Python, while the foregoing significant correlation is not seen for the students learning Java. According to the results of the t-test analysis, the learning achievement of the students learning Python is significantly higher than that of the students learning Java, which indicates that for students, learning Python has better learning motivation and self-efficacy perception, and reduces their maladaptive cognition to obtain better learning achievement.

## Conclusion

This study used an experimental design to compare students’ learning performance and effectiveness in Python and Java programming courses. The experimental results showed that students’ learning effectiveness, learning motivation, computer programming self-efficacy, and maladaptive cognitions could be significantly improved in the Python programming class. Possible reasons are that Python is simpler in data and prograaming structure and its syntax is shorter (Khoirom et al., 2020). The results of the research can be a reference for programming teaching/learning. However, this study still has some limitations. First of all, the experiment is conducted alongside the courses’ teaching, the courses given in this study take a longer time, and the number of classes is fewer, resulting in insufficient samples. In the future, more questionnaires are expected to be collected from students of more classes of the foregoing two programming languages to increase the number of subjects under test for further exploration and study. Secondly, in the future, more diversified programming language courses are expected to be studied at the same time to understand the impacts of different programming languages on students’ learning performance, to find out the programming languages more suitable for students to learn. This study also explores the factors that affect the learning performance of different programming languages based on students’ learning motivation, self-efficacy, and the negative factor, maladaptive cognitions. In terms of the exploration of the factors affecting the learning performance of different programming languages, it is recommended to explore the impact of students’ satisfaction with the content of the programming language course on their learning performance and to examine whether students’ experience about the content of different programming language courses may affect their learning performance.

## Data Availability Statement

The raw data supporting the conclusions of this article will be made available by the authors, without undue reservation.

## Ethics Statement

The studies involving human participants were reviewed and approved by Teaching Practice Research Program, Ministry of Education, Republic of China (Taiwan). The patients/participants provided their written informed consent to participate in this study.

## Author Contributions

H-CL contributed to the research topic and the methodology. K-LH contributed to the research model and the experimental design and results. W-CH contributed to the statistical analysis and the discussion. All authors contributed to the article and approved the submitted version.

## Conflict of Interest

The authors declare that the research was conducted in the absence of any commercial or financial relationships that could be construed as a potential conflict of interest.
